# Neuroplasticity and functional reorganization of language in patients with arteriovenous malformations: insights from neuroimaging and clinical interventions

**DOI:** 10.3389/fnhum.2025.1503864

**Published:** 2025-01-31

**Authors:** Jialong Yuan, Hongchuan Niu, Chengxu Lei, Ruichen Xu, Yutong Liu, Kexin Yuan, Linru Zou, Shihao He, Yuanli Zhao

**Affiliations:** ^1^Department of Neurosurgery, Peking Union Medical College Hospital, Peking Union Medical College and Chinese Academy of Medical Sciences, Beijing, China; ^2^Department of Neurosurgery, Peking University International Hospital, Beijing, China

**Keywords:** arteriovenous malformation, functional speech area, reorganization of speech function, functional magnetic resonance imaging, targeted preoperative assessments

## Abstract

Patients with arteriovenous malformations (AVMs) located in the functional area of speech often exhibit language dysfunction, and neuroplasticity allows the brain of some patients to regain speech through functional reorganization. Exploring the mechanism of AVMs-induced reorganization of language function is important for understanding neuroplasticity and improving clinical intervention strategies. This review systematically searched and analyzed the research literature in related fields in recent years, covering data from neuroimaging, functional magnetic resonance imaging (fMRI), and clinical case studies. By integrating these evidences, the phenomenon of functional reorganization within non-verbal functional areas and its influencing factors in patients with AVMs were assessed. It concluded that functional reorganization of language due to AVMs is a manifestation of a high degree of neurological plasticity and that understanding this process has important implications for neurosurgical planning and postoperative rehabilitation of patients. Future research should continue to explore the mechanisms of functional reorganization in the brain and work to develop new diagnostic tools and therapeutic approaches to improve the rate of recovery of language function in patients with AVMs.

## 1 Introduction

Cerebral arteriovenous malformations (AVMs) typically manifest as haemorrhage (50%), epilepsy (25%), and headache (15%) (Cirillo et al., 2019; Gajardo-Vidal et al., 2021; Wilson et al., 2015), and are characterized by blood-supplying arteries, malformed blood vessel clusters, and draining veins (Friedlander, 2007; Teter and Sawitzke, 2017). Linguistic function areas are the key areas in the brain responsible for language processing, mainly involving Broca’s, Wernicke’s, Geschwind’s areas, which are interconnected by a complex neural network to form a language processing network (Nasios et al., 2019). The current mainstream view is that language formation involves two pathways: the dorsal and ventral pathways (Zhang et al., 2018; Hagoort, 2019; Chang et al., 2015). Major fascicles in the dorsal pathway were superior longitudinal fasciculus (SLF) and arcuate fasciculus (AF). The ventral pathway predominantly consists of the anterior thalamic radiation (ATR), inferior longitudinal fasciculus (ILF), inferior frontal-occipital fasciculus (IFOF) and the uncinate fasciculus (UF) (Hagoort, 2019). All these fiber bundles participate in language processing and play different roles. These regions communicate through complex neural networks to coordinate the functions of speech production, comprehension, memory, and communication. Due to the anatomical location of the functional areas of speech, these areas have a high demand for localized blood flow, which means that any vascular abnormality, such as an AVMs, can adversely affect speech function (Fridriksson et al., 2018). An AVMs situated in these areas may lead to speech impairment due to the compression or destruction of normal nerve tissue. Additionally, AVMs can lead to localized bleeding, worsening nerve damage and resulting in more severe speech dysfunction.

The effects of AVMs on language function are not limited to anatomical changes, but may involve a more complex reorganization of neural networks. There have been some suggestions that the formation of AVMs in the brain and damage to “hypothetical” language areas should be earlier than the formation of “real” language areas (Deng et al., 2015). Patients with AVMs involving the language area can be considered as models of congenital “knock-out” language area, but these patients nevertheless acquire normal language function during subsequent language learning (Cirillo et al., 2019; Gajardo-Vidal et al., 2021; Ille et al., 2018). It is postulated that this feature is analogous to the functional reorganization observed in functional area gliomas. In contrast to acute stroke events, acute stroke resulting in aphasia is typically caused by acute cerebral ischemic necrosis, which often has a poor prognosis. If the embolism area is extensive, the damage to the language pathway is irreparable, and the time required to regain language function is considerable. In contrast, aphasia caused by glioma or AVMs is also a chronic compression process. When the compression reaches the language conduction pathway, the functional reorganization process has already begun, which can facilitate the patient’s recovery of language function as soon as possible after surgery or other treatments. Therefore, the mechanism of language reorganization in AVMs patients may be different from that in acquired disorders. In some cases, recovery of language function relies on bilateral brain cooperation, especially when the left language area is severely damaged and the right brain becomes more involved in language tasks (Lee et al., 2010). fMRI studies have also shown increased activation of the right inferior frontal gyrus and other related areas, which may be a marker of functional reorganization (Deng et al., 2015; Deng et al., 2019). Although this reorganization provides important information for preoperative assessment, its clinical application remains challenging.

Understanding the mechanisms of functional reorganization of language in patients with AVMs is important for the development of individualized surgical and rehabilitation plans that can help neurosurgeons effectively manage the deformity while maximizing preservation of the patient’s language function. This review provides a comprehensive overview of the existing research on ectopic reorganization of speech function in AVMs in the area of speech function.

## 2 Effect of AVMs on the functional speech area

### 2.1 Altered speech function due to AVMs

When an AVMs is located in a functional area of the brain related to language, patients may experience a wide range of symptoms of language disorders. The severity and type of symptoms depend on the specific location and size of the AVMs and its effect on the surrounding brain tissue. Common symptoms include: Aphasia, which is the loss of the ability to express or understand language, or varying degrees of reduced fluency and difficulty using vocabulary. The specific area of language function where the AVMs is located determines the type and severity of aphasia. If the AVMs is located in Broca’s area, the patient may experience expressive aphasia, which manifests as difficulty with language production (Flinker et al., 2015; Li et al., 2020). The patient is usually able to understand language, but has difficulty expressing his or her thoughts fluently. Similarly, if the AVMs is located in Wernicke’s area, patients may experience perceptual aphasia, which is a difficulty with language comprehension. The content of the patient’s speech may be meaningless or difficult to understand, even though the patient is able to speak fluently. In addition, an AVMs located between these major areas of language function may result in mixed aphasia, which manifests as symptoms of both expressive and comprehension difficulties.

### 2.2 Effects of AVMs on hemodynamics in functional speech areas

The impact of AVMs on speech function is not only due to their occupying effect and direct tissue destruction, but also involves complex hemodynamic alterations leading to significant changes in blood supply to local brain tissues through abnormal arteriovenous shunts (Chen et al., 2020). The presence of an AVMs could result in reduced blood flow to the surrounding brain tissue, which may be worsened by the “steal phenomenon, “where the AVMs diverts blood away from the surrounding tissue due to abnormally high flow rates (Spetzler et al., 1992; Norris et al., 1999; Moftakhar et al., 2009). This condition may be exacerbated by the “steal phenomenon, “in which the AVMs “steals” blood supply from the surrounding normal brain tissue through abnormally high blood flow rates. This abnormal blood flow distribution results in a relatively low oxygen and nutrient status of the normal brain tissue, especially the language function area, affecting its normal function (Mast et al., 1995; Fierstra et al., 2011; Kim and Krings, 2011). Moreover, the occurrence of haemorrhage from the AVMs is a significant contributor to the impairment of speech function. This is particularly pronounced in instances of massive haemorrhage leading to widespread brain damage, where patients may suddenly and severely lose their ability to speak. In a study of patients with AVMs in language areas, researchers found that while some patients maintained relatively normal language function in the presence of an AVMs, these patients tended to experience significant language dysfunction after surgical treatment or rupture of the AVMs. This suggests that AVMs may progressively affect speech function through slowly progressive hemodynamic changes, but that this effect is rapidly exacerbated when stimulated by external factors. However, contrary results have been obtained in studies in which, in some patients with hemorrhagic AVMs located in the frontal lobe, complete surgical resection of the responsible lesion resulted in the disappearance of the postoperative aphasia and complete restoration of speech function (Kunc, 1974).

### 2.3 Effects of AVMs with different endings on speech reconstruction

The recovery and reconstruction process of speech dysfunction caused by cerebral AVMs varies according to two different scenarios: acute haemorrhage and slow enlargement of the lesion. Prior to the onset of the haemorrhage, the AVMs did not appear to affect the organization of language function, which was located exclusively in the left hemisphere. Some believe that congenital asymptomatic AVMs lesions in the speech area may primarily damage the myelin, with no axonal involvement in most patients. This may result in relatively little damage to speech-related pathways and preservation of their critical functions, leaving patients with intact speech function (Teter and Sawitzke, 2017; Deng et al., 2015; Chen et al., 2020; Deng et al., 2022).

In another study, researchers found that language dysfunction may occur rapidly after acute haemorrhage in AVMs due to haematoma compression or brain tissue damage, and that language activation may be observed not only *in situ*, but also in the contralateral hemisphere even if the patient’s language function has been fully restored (La Piana et al., 2009). This phenomenon may indicate that normal language function after haemorrhage requires support from both hemispheres.

### 2.4 Basis of neuroplasticity and brain reorganization

Neuroplasticity is the brain’s nervous system’s ability to adapt dynamically in terms of both structure and function. It holds significant importance in the realms of learning, memory, and rehabilitation following neurological injury. Recent studies have indicated that neuroplasticity is governed by several molecular mechanisms. These encompass Cortical plasticity, regulation of brain-derived neurotrophic factor (BDNF), and cellular signaling pathways.

#### 2.4.1 Functional remodeling of the right hemisphere

Cortical functional remodeling is an important phenomenon in patients with cerebral AVMs, manifesting as a transfer of important neurological functions from affected brain regions to adjacent uninvolved brain regions due to chronic vascular steal caused by the AVMs (Moftakhar et al., 2009). This process is dependent on the neuroplasticity of the brain and is influenced by the location of the AVMs and therapeutic interventions. Current studies mostly use functional MRI and other methods to assess cortical functional remodeling (Jiao et al., 2021). Synaptic plasticity is at the heart of cortical plasticity and is achieved primarily through two forms of long-term potentiation (LTP) and long-term depression (LTD). LTP is a sustained increase in the efficiency of synaptic transmission in response to radiofrequency stimulation and is typically based on the activation of NMDA (N-methyl-D-aspartic acid receptor) receptors. The permeability of the NMDA receptor to calcium ions allows the influx of calcium ions into the cell, which activates a number of downstream signaling pathways, resulting in the endogenous insertion of AMPA (*α*-amino-3-hydroxy-5-methyl-4-isoxazole-propionic acid) receptors and increased responsiveness of the postsynaptic membrane. In contrast, LTD exhibits a long-term reduction in synaptic transmission efficiency. This is typically associated with low-frequency stimulation (Luscher and Malenka, 2012). These mechanisms offer the physiological foundation for the process of learning and memory. However, there are limitations in the current study due to the scarcity of data and the variety of assessment methods.

**Figure 1:**
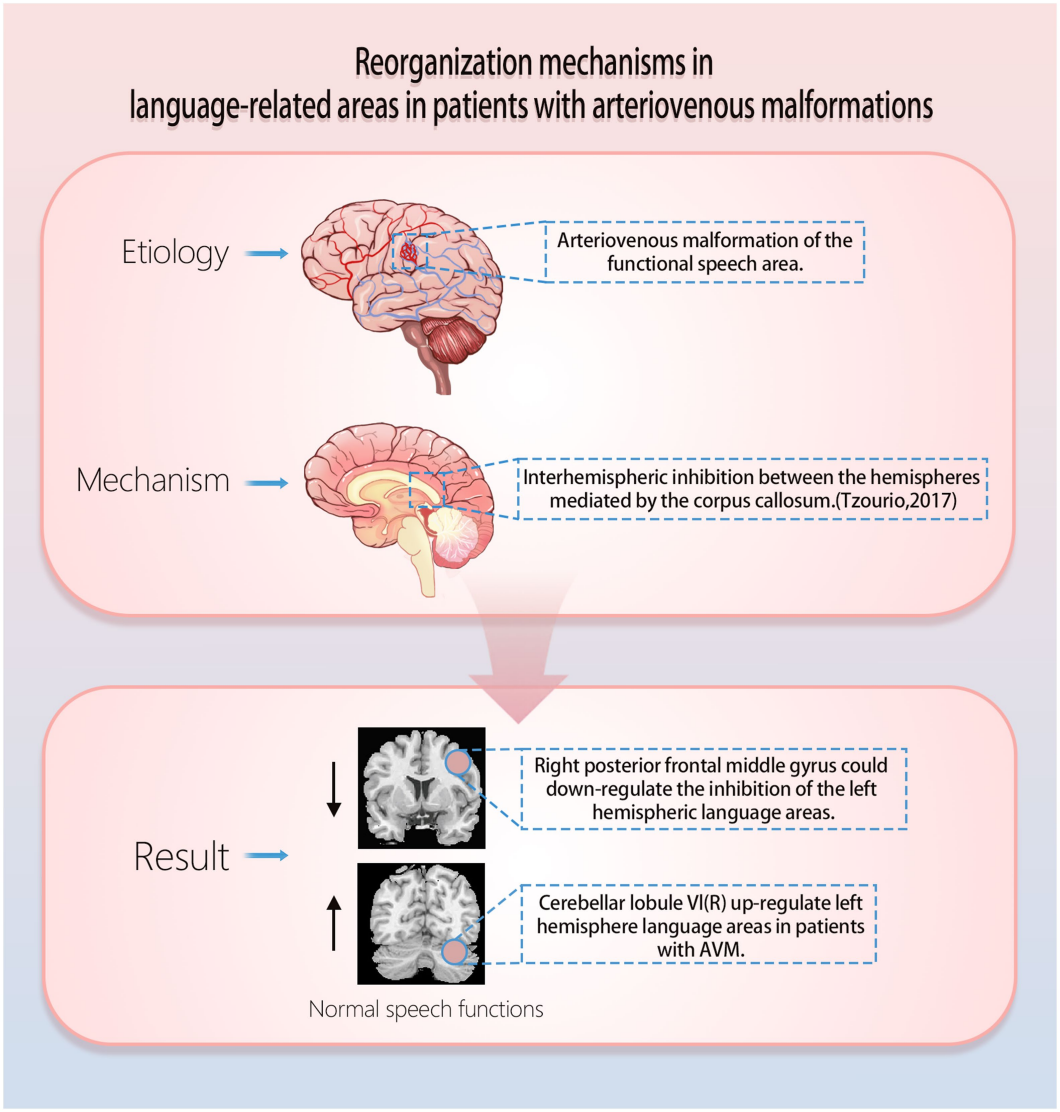
Mechanisms of functional reorganization of arteriovenous malformations in speech function.

Typically, for most people language areas are located in the left cerebral hemisphere, and recruitment of right hemispheric language function may occur when AVMs are present in the left cerebral hemisphere language function area (Figure 1).

It has been found that AVMs lesions that involve traditional language areas (left hemisphere language areas) show significantly enhanced activation of the patient’s right hemisphere anterior central gyrus (BA6) and the cerebellar right lobule VI in an auditory sentence comprehension task (Deng et al., 2021). Specifically, the right cerebellar lobule VI may support the maintenance of language function by upregulating the function of damaged language regions in the left hemisphere, whereas the right precentral gyrus may support the maintenance of language function by reducing interhemispheric inhibition (Figure 1; Deng et al., 2021; Tzourio-Mazoyer et al., 2017; McAvoy et al., 2016; Chu et al., 2018; Hartwigsen and Saur, 2019). In a study published in Stroke, it was found that the left temporal AVMs impairs IFOF and ILF (ventral pathways), and that ventral pathways in the right hemisphere (ATR and UF) may be more important for reorganization; the left parietal AVMs impairs the left AF, IFOF, and ILF (dorsal and ventral pathways), and that the reorganised ventral pathways (right ATR, UF, and right IFOF) may be involved in the reconstruction of new language networks (Deng et al., 2022). In a study of gliomas of the left language function area, Kinno et al. found a dual pathway between the left PLIC (posterior limb of the internal capsule) and the right PI (posterior insula), which may be the mechanism for the presence of normal language in patients with damage to either pathway (Kinno et al., 2023). In addition, both pathways are connected through the corpus callosum (Kinno et al., 2014; Glasser et al., 2016), and it is possible that the right PI functions as a new incumbent language function area and also upregulates the function of the left hemisphere residual language function area through the corpus callosum. This helps to explain why some patients experience acute postoperative language deficits after removal of a functional area of language, while others recover gradually. This helps to explain the mechanism of language reorganization. These findings not only enhance our understanding of how the AVMs affects language networks, but also provide new perspectives for exploring the effects of congenital cerebrovascular lesions on brain plasticity.

#### 2.4.2 Cell signaling pathway

Cellular signaling pathways are also critical in neuroplasticity, especially the mammalian target of rapamycin (mTOR) and MAPK/ERK pathways. The mTOR signaling pathway governs the synthesis of proteins and the proliferation of cells by combining signals from nutrient levels, energy availability, and growth factors (Rose and Cathey, 2022). In neurons, activation of mTOR not only promotes synapse formation but also plays a key role in memory consolidation. In addition, the MAPK/ERK signaling pathway further affects synaptic plasticity and neural adaptation by regulating gene expression and its associated protein synthesis (Nikolaev et al., 2018).

#### 2.4.3 Complexity of integration mechanisms

These molecular mechanisms do not exist independently but are a complex network of multiple signaling pathways interacting to regulate neuroplasticity. For example, the establishment of LTP is dependent on the intervention of BDNF (Leal et al., 2014), while synaptic plasticity is achieved through mTOR and MAPK/ERK signaling pathways (Giachello et al., 2010). Thus, neuroplasticity can be viewed as a multilevel, multidimensional biological process involving interactions at multiple levels of cell signaling, gene expression regulation, and protein synthesis.

### 2.5 Relationship between age and neuroplasticity

In terms of neuroplasticity and functional reorganization, age is an important factor. Studies have shown that younger patients have greater brain plasticity (Guo and Zhao, 2011). This means that their brains are able to reorganize functionally more efficiently after damage to functional areas of language (Smith, 2013). This may be due to the fact that younger patients have more flexible neural networks. These networks can adjust and reconfigure rapidly in order to meet new functional requirements. In contrast, the brains of older patients have less plasticity and therefore tend to be less effective at re-organizing following AVMs-induced language damage. This has been demonstrated in numerous clinical studies. Many of these studies have shown that younger patients have faster and better recovery of language function, whereas older patients may require longer rehabilitation and have more limited recovery (Burke and Barnes, 2006; Goldsworthy et al., 2020; Nicolini et al., 2021).

## 3 Clinical interventions and prognosis

### 3.1 Pre-operative assessment and planning

fMRI is one of the core techniques used to assess preoperative functional speech areas. fMRI creates a real-time visualization of brain activity by monitoring fluctuations in blood oxygen level-dependent (BOLD) signals while the brain engages in specific activities, like speaking (Deng et al., 2021). This technique allows the surgeon to assess the location, extent, and relative activity of functional language areas preoperatively, avoiding interference with these critical areas during surgical planning. fMRI can reveal specific patterns of activation in language areas during language-related tasks (such as naming, reading, or semantic judgments), which typically include the left inferior frontal gyrus and left superior temporal gyrus (Cannestra et al., 2004). By examining these regions of activation, surgeons can enhance their comprehension of the patient’s language function organization and establish a personalized surgical strategy, ultimately minimizing the surgery’s impact on language function (Mahvash et al., 2014).

Diffusion tensor imaging (DTI) is a critical imaging method that reveals the structural connections between different areas of the brain by visualizing white matter fiber bundles. It is especially effective in illustrating the principal white matter pathways linking language-related regions, such as the arcuate fasciculus—a crucial tract connecting Broca’s area and Wernicke’s area (Li et al., 2020; Itoh et al., 2006). Preoperative DTI images can show the distribution of patient-specific white matter fiber bundles, thus guiding the design of surgical pathways to avoid damage to these language-related pathways. This imaging technique not only helps to prevent postoperative language dysfunction, but also provides valuable reference information for postoperative rehabilitation. It is important to remember that the specific localization of functional language areas can vary greatly between individuals. Traditionally, functional areas of language are thought to be located predominantly in the left hemisphere, but in some individuals (especially left-handed or ambidextrous individuals), language function may show different patterns of organization (Li et al., 2021). For example, speech function may be partially or completely distributed in the right hemisphere, and this individual variation makes accurate preoperative assessment particularly important. With individualized assessment by fMRI and DTI, surgeons can accurately identify the unique distribution of speech function in each patient and avoid unintentional injury during surgery (Deng et al., 2021). In addition, to better characterise white matter (WM) pathway integrity and tract orientations, analytical and modelling techniques such as automated fiber quantification (AFQ) (Yeatman et al., 2012) have been developed. As a fully automated approach, AFQ allows reliable identification of major WM pathways, facilitating further quantitative and statistical analysis of pathways at anatomically equivalent sites. Complete visualization and quantification of whole-brain major fibres in language-involving AVMs patients may shed light on language impairment and plasticity mechanisms during disease development from birth (Deng et al., 2022).

### 3.2 Rehabilitation and functional restoration

Postoperative rehabilitation is an important stage in the functional reorganization and restoration of language function in patients with AVMs within the area of speech function (Clinchot et al., 1994). Successful rehabilitation strategies rely not only on early intervention and systematic rehabilitation programs but also on advanced neuromodulation techniques to facilitate reorganization and recovery of brain function. Key aspects of rehabilitation and functional recovery, including traditional speech therapy and noninvasive brain stimulation techniques are explored in detail below.

#### 3.2.1 Traditional speech therapy

Traditional speech therapy is the most basic and core part of the rehabilitation process, aiming to help patients regain their lost language skills through systematic language training and practice. Speech and language therapists will design an individualized treatment plan based on the patient’s specific language impairment, covering phonology, vocabulary, grammar, and pragmatics. Research has shown that early and consistent speech therapy can significantly improve a patient’s language functioning (Stephens, 2001; Galletta and Barrett, 2014).

#### 3.2.2 Noninvasive brain stimulation techniques

In recent times, there has been a growing interest in the use of noninvasive brain stimulation techniques like transcranial magnetic stimulation (TMS) and transcranial direct current stimulation (tDCS) for the purpose of restoring language function. These techniques speed up functional reorganization and recovery by modulating cortical excitability and promoting neuroplasticity.

TMS, a technique that uses magnetic fields to stimulate the cerebral cortex, has been shown to have the potential for facilitating the recovery of language function demonstrated that stimulation of the Broca’s area induced adaptive reorganization of the right homologous area, thereby supporting the recovery of language function after surgery (Hartwigsen, 2015; Zhang et al., 2017).

tDCS, which regulates the activity of the cerebral cortex by applying a weak direct current, has been applied to the rehabilitation of language function. Enhancing the plasticity of language regions in the left hemisphere, promotes the formation of new neural connections, thereby accelerating the reorganization and recovery of language function. In addition, it was discovered that tDCS effectively suppressed overactive homologous regions, decreasing their detrimental effects on language function recovery and promoting the redistribution and optimization of brain function (Silva et al., 2018; You et al., 2011; Kang et al., 2011).

The presence of AVMs affecting speech function poses a significant threat to patients “ability to speak.” However, there is hope for preserving or restoring speech function in these patients through mechanisms of neuroplasticity and functional reorganization. This article reviews functional reorganization, preoperative assessment and planning, clinical intervention, and prognosis in the functional domain of language. In the concluding section, we will present some possible future directions and reasonable ideas considering the latest advances in current technological and medical developments.

As machine learning and big data analytics become more prevalent in medicine, we foresee the creation of predictive models that rely on individual patient characteristics such as age, sex, genotype, and neural network structure. These models could help surgeons better predict which patients are most likely to benefit from preoperative functional reorganization and develop more targeted preoperative assessments and postoperative rehabilitation plans (Subrahmanya et al., 2022).

Current fMRI and DTI techniques have already provided us with a basic understanding of patients with AVMs in the functional areas of language, but these techniques still have some limitations, such as limited resolution and insufficient sensitivity to hemodynamic changes. In the future, ultra-high-resolution fMRI and enhanced DTI techniques may provide a more fine-grained map of brain region function and connectivity, helping surgeons to more clearly identify and protect functional areas of language. Moreover, cutting-edge technologies like functional near-infrared spectroscopy (fNIRS) (Asgari et al., 2003; Keuler et al., 2018) can monitor neural activity in real-time and offer immediate intraoperative feedback to dynamically refine surgical approaches. Integration of multimodal imaging techniques will become part of routine clinical practice. For example, combining fMRI with electrophysiological monitoring (Chang et al., 2016) enables simultaneous observation of functional brain activity in both spatial and temporal dimensions. The application of such multimodal imaging will not be limited to preoperative assessment but can also be extended to postoperative rehabilitation for monitoring the functional reorganization process and adjusting the rehabilitation plan.

Personalized medicine is an important direction in the development of modern medicine, especially when dealing with complex neurological disorders such as AVMs, personalized treatment and rehabilitation programs are especially critical. With advances in genomics (Florian et al., 2020), epigenetics (Florian et al., 2020), neuroimaging (Di Ieva et al., 2015), and other fields, we can hope to customize treatment plans for each patient in the future. A plausible scenario is to use artificial intelligence (AI) and machine learning technologies to develop personalized rehabilitation programs. These AI systems can analyze the patient’s neuroimaging data in real-time and adjust the difficulty and type of rehabilitation tasks according to the progress of functional reorganization, thus providing more precise rehabilitation training. In addition, virtual reality (VR) technology may also play an important role in future rehabilitation training by providing immersive and more interactive language training environments to facilitate reconnection of neural networks and functional recovery (Ruparelia et al., 2023).

Despite the valuable insight gained from the current study on functional reorganization in AVMs patients, there remain numerous unanswered questions requiring further detailed investigation. In the future, the primary focus of research will be on comprehending the molecular and cellular mechanisms of functional reorganization. This will not just help to clarify the foundation of neuroplasticity. It will also establish a theoretical foundation for the advancement of innovative therapeutic strategies. Another important direction is to investigate how external stimulation (transcranial magnetic stimulation TMS or transcranial direct current stimulation tDCS) (Silva et al., 2018; You et al., 2011; Kang et al., 2011) can enhance the efficiency of functional reorganization. There is initial evidence suggesting that these techniques may help enhance the recovery of language function. Nevertheless, the mechanisms of action remain incompletely understood.

### 3.3 Limitation

This article systematically reviews the effects of AVMs on areas of language function and the role of neuroplasticity and functional reorganization. There are several limitations to the article, although it provides valuable insights into understanding the effects of AVMs on language function. First, the generalizability and accuracy of the results may be affected by limitations in the references cited. Second, the resolution and sensitivity of current imaging modalities, such as fMRI and DTI, need to be improved to better assess changes in functional areas of speech. In addition, in-depth exploration of potential therapeutic strategies is limited by a lack of understanding of the specific molecular and cellular mechanisms of neuroplasticity and functional reorganization. Finally, the diversity of clinical interventional strategies and the uncertainty regarding rehabilitation outcomes are also challenging. We hope that future studies will further overcome these limitations to advance the field.

## 4 Conclusion

Overall, treating and rehabilitating AVMs in the area of language function presents complex challenges, but is also full of potential opportunities. In the upcoming years, advancements in neuroscience, imaging technology, and personalized medicine are anticipated to enable more accurate surgical planning and rehabilitation training, leading to substantially enhanced patient outcomes. Future research should continue to explore the mechanisms of neuroplasticity and functional reorganization in depth. Combined with the application of new technologies, this will provide better medical solutions for AVMs patients.
